# Survey of Pathogen-Lowering and Immuno-Modulatory Effects Upon Treatment of *Campylobacter coli*-Infected Secondary Abiotic IL-10^−/−^ Mice with the Probiotic Formulation Aviguard^®^

**DOI:** 10.3390/microorganisms9061127

**Published:** 2021-05-23

**Authors:** Dennis Weschka, Soraya Mousavi, Nina Biesemeier, Stefan Bereswill, Markus M. Heimesaat

**Affiliations:** Gastrointestinal Microbiology Research Group, Institute of Microbiology, Infectious Diseases and Immunology, Charité—Universitätsmedizin Berlin, Corporate Member of Freie Universität Berlin, Humboldt-Universität zu Berlin, and Berlin Institute of Health, 12203 Berlin, Germany; dennis.weschka@charite.de (D.W.); nina.biesemeier@charite.de (N.B.); stefan.bereswill@charite.de (S.B.)

**Keywords:** competitive exclusion product, Aviguard^®^, enteropathogenic infection, *Campylobacter coli*, immune-modulatory effects, secondary abiotic IL-10^−/−^ mice, campylobacteriosis model, host–pathogen-interaction, probiotic formulations

## Abstract

The prevalence of infections with the zoonotic enteritis pathogen *Campylobacter coli* is increasing. Probiotic formulations constitute promising antibiotic-independent approaches to reduce intestinal pathogen loads and modulate pathogen-induced immune responses in the infected human host, resulting in acute campylobacteriosis and post-infectious sequelae. Here, we address potential antipathogenic and immuno-modulatory effects of the commercial product Aviguard^®^ during experimental campylobacteriosis. Secondary abiotic IL-10^−/−^ mice were infected with a *C. coli* patient isolate on days 0 and 1, followed by oral Aviguard^®^ treatment on days 2, 3 and 4. Until day 6 post-infection, Aviguard^®^ treatment could lower the pathogen burdens within the proximal but not the distal intestinal tract. In contrast, the probiotic bacteria had sufficiently established in the intestines with lower fecal loads of obligate anaerobic species in *C. coli*-infected as compared to uninfected mice following Aviguard^®^ treatment. Aviguard^®^ application did not result in alleviated clinical signs, histopathological or apoptotic changes in the colon of infected IL-10^−/−^ mice, whereas, however, Aviguard^®^ treatment could dampen pathogen-induced innate and adaptive immune responses in the colon, accompanied by less distinct intestinal proinflammatory cytokine secretion. In conclusion, Aviguard^®^ constitutes a promising probiotic compound to alleviate enteropathogen-induced proinflammatory immune responses during human campylobacteriosis.

## 1. Introduction

*Campylobacter* infections are the leading causes of bacterial gastroenteritis in humans, with worldwide rising prevalences [1]. In the European Union, for instance, campylobacteriosis was the most frequent zoonotic disease in 2018, responsible for more than 240,000 reported cases. *Campylobacter jejuni* and *Campylobacter coli* constituted the most prevalent causative agents with relative abundances of 83.9% and 10.3%, respectively [2]. The curved or rod-shaped and highly motile Gram-negative bacteria are part of the commensal gut microbiota of many avian species, including chicken and turkey and of mammals, such as pigs, cattle and sheep and exhibit a growth optimum at 37–42 °C [3,4]. In addition, *Campylobacter* species might be isolated from natural environments, including surface waters and can survive extended periods outside a warm-blooded host [4,5]. The bacteria are mainly transmitted to humans via the food chain. Ingestion of undercooked or raw meat products from livestock, contaminated water, eggs or unpasteurized milk is a potential infection source. However, most *Campylobacter* outbreaks result from ingestion of poultry products and/or inappropriate handling and kitchen hygiene measures [6,7]. Of note, a European survey from 2018 revealed a *Campylobacter* prevalence of 26% in broiler chicken and of 72% in turkeys [8]. In slaughterhouses, *Campylobacter* has been shown to spread from the ceca of infected animals, subsequently contaminating the entire carcass [9,10]. Importantly, *Campylobacter* was not only isolated from the meat of *Campylobacter* colonized chicken but also from secondarily cross-contaminated chickens whose feces had initially been *Campylobacter*-negative [11]. Physical, chemical and radiation-based methods are generally applied to eliminate *Campylobacter* species from livestock carcasses and slaughterhouse equipment. However, those procedures may significantly reduce the *Campylobacter* loads but not eliminate the contaminating agents [11,12,13]. Notably, *Campylobacter* species can be highly infectious, given that relatively low doses of 500 bacteria may already lead to campylobacteriosis in infected humans depending on both the arsenal of virulence factors expressed by the pathogen and on the host immune status [5,14]. After an incubation period of up to five days, *Campylobacter* infected patients may present with symptoms of varying severities ranging from mild malaise to acute campylobacteriosis characterized by fever, chills, abdominal cramps, headache, and watery or even bloody diarrhea with mucous discharge and inflammatory cells due to underlying enterocolitis [15,16]. Notably, from the severity of the induced campylobacteriosis syndrome, one cannot predict the respective causative *Campylobacter* species [6]. The disease usually only requires symptomatic treatment, such as the substitution of fluids and minerals and resolves within two weeks post-infection (p.i.) without residues [14,15,16]. Post-infectious autoimmune morbidities affecting the nervous system (e.g., Guillain–Barré or Miller–Fisher syndrome), the joints (i.e., reactive arthritis) and the gastrointestinal tract (i.e., inflammatory bowel disease and irritable bowel syndrome), however, may develop in rare occasions weeks to months p.i. [16,17,18]. Particularly sialylated lipooligosaccharide (LOS) derived from the cell wall of Gram-negative bacterial species, including *Campylobacter*, is associated with severe forms of campylobacteriosis and increased risk of post-infectious manifestations [19].

Almost 40 years ago, distinct probiotic formulations were hypothesized as promising tools to exclude potentially pathogenic bacteria from their ecological niches within the vertebrate gastrointestinal tract [20,21]. Commercial competitive exclusion products have gained access to the agricultural industry in the meantime and have been successfully applied as antibiotic-independent animal feed additives to reduce the prevalence of enteropathogens, such as *Salmonella* and *Clostridium perfringens* in livestock and subsequently, to decrease the prevalence of foodborne infections in humans [22,23,24,25]. Among these commercial exclusion products, Aviguard^®^ constitutes a mixture of viable probiotic bacteria representative for the main commensal species within the cecum of adult chicken [24]. This freeze-dried fermentation product exhibits a longer shelf life when compared to other compounds. It is applied as a drinking water additive or spray treatment to poultry [24].

To date, however, studies elucidating the triangle interplay between *Campylobacter*, probiotic bacteria derived from a commercial exclusion product including Aviguard^®^ and the mammalian host are scarce. This prompted us in our present study for the first time to investigate the potential pathogen-lowering effects and immune-modulatory properties of peroral Aviguard^®^ treatment during experimental campylobacteriosis. We, therefore, applied a murine *Campylobacter*-induced infection and inflammation model that had initially been generated to dissect *C. jejuni*-host interactions. Following peroral infection of secondary abiotic *interleukin 10* gene-deficient (IL-10^−/−^) mice that had been generated following broad-spectrum antibiotic treatment, *C. jejuni* could not only stably infect the murine gastrointestinal tract but also induced non-self-limiting acute enterocolitis within a week p.i. [26]. The underlying mechanisms for this inflammatory scenario are pronounced Toll-like receptor-4 (TLR-4)-dependent, LOS host immune responses affecting not only the intestinal tract but also extra-intestinal, including systemic body parts [26,27]. Very recently, the secondary abiotic IL-10^−/−^ mouse infection and inflammation model has been proven suitable also to unravel *C. coli*-host interactions [28,29]. In our actual study, we infected secondary abiotic IL-10^−/−^ mice with a *C. coli* isolate derived from a diseased human patient by gavage, followed by oral Aviguard^®^ treatment on three consecutive days, and surveyed (i.) the intestinal pathogen loads over time, (ii.) the clinical and microscopic inflammatory changes, and (iii.) the proinflammatory immune responses in intestinal and systemic compartments upon sacrifice at day 6 p.i.

## 2. Materials and Methods

### 2.1. Ethical Statement

All murine experiments had initially been approved by the commission for animal experiments headed by the “Landesamt für Gesundheit und Soziales” (LaGeSo, Berlin; registration number G0172/16) and were performed according to the European Guidelines for animal welfare (2010/63/EU) and the ARRIVE guidelines. Clinical conditions of mice were monitored daily.

### 2.2. Secondary Abiotic IL-10^−/−^ Mice

IL-10^−/−^ mice (C57BL/6j background) were bred in the Forschungsinstitute für Experimentelle Medizin, Charité—University Medicine Berlin (Berlin, Germany) and maintained in sterile cages covered by filter tops within an experimental semi-barrier facility. By the age of 3 weeks, mice were subjected to an 8-week-course of broad-spectrum antibiotic treatment for commensal gut microbiota depletion as described in detail elsewhere [30,31]. The secondary abiotic status of mice was confirmed by both culture and culture-independent (i.e., molecular, 16S rRNA-based) methods, as reported earlier [30,32]. Microbiota-depleted mice received autoclaved drinking water and chow and were handled under aseptic conditions to avoid contaminations. For antibiotic washout, the antibiotic cocktail was replaced by autoclaved tap water as early as three days before infection.

### 2.3. Campylobacter Coli Infection

The *C. coli* isolates (from the stool of a diseased patient with bloody diarrhea) were kindly provided by Dr. Torsten Semmler (Robert-Koch-Institute Berlin, Germany). Two days before infection, the *C. coli* isolate was thawed from stock and cultivated on Columbia agar (supplemented with 5% sheep blood) and Karmali agar plates (both from Oxoid, Wesel, Germany) that were incubated in a jar containing CampyGen gas packs (Oxoid, Wesel, Germany) under microaerophilic conditions (37 °C, 48 h). On days 0 and 1, sex- and age-matched secondary abiotic IL-10^−/−^ mice (11 week-old litter mates, balanced gender ratio) were challenged with 10^9^ colony-forming units (CFU) *C. coli* by gavage.

### 2.4. Treatment of Mice With the Commercial Exclusion Product Aviguard^®^

For probiotic treatment, 1 g Aviguard^®^ (Lallemand Animal Nutrition, Worcestershire, UK) was dissolved in 10 mL phosphate-buffered saline (PBS; Thermo Fisher Scientific, Waltham, MA, USA) and perorally applied to mice on days 2, 3 and 4 p.i. (0.3 mL by gavage). The competitive exclusion product consists of the following bacterial species (approximately 10^9^ CFU per mL): *Escherichia coli*, *Citrobacter* species, *Enterococcus* genus (*E. faecalis*, *E. faecium*), *Lactobacillus* species (*L. casei*, *L. plantarum*), *Bacteroides* species, *Clostridium* species (*C. sporogenes*), *Eubacterium* species, *Propionibacterium* species, *Fusobacterium* species, *Ruminococcus* species [33]. Placebo-treated *C. coli*-infected mice received vehicle only (0.3 mL by gavage). Naive control animals were neither infected with *C. coli* nor challenged with Aviguard^®^ nor placebo.

### 2.5. Gastrointestinal C. coli Loads

For quantification of the gastrointestinal *C. coli* burdens, fecal and luminal stomach, duodenum, ileum, and colon samples were homogenized and serial dilutions streaked onto Columbia agar plates supplemented with 5% sheep blood and onto selective Karmali plates (both from Oxoid, Wesel, Germany). The inoculated plates were incubated in a jar containing CampyGen gas packs (Oxoid, Wesel, Germany) under microaerophilic conditions (37 °C, 48 h). *C. coli* were identified by the distinct macroscopic morphotypes, microscopic appearance following Gram-staining, and oxidase-positive reaction.

### 2.6. Cultural Intestinal Microbiota Analysis

To provide a comprehensive quantitative cultural survey of the bacterial compositions in both the Aviguard^®^ suspensions and murine feces, respective samples were homogenized in sterile phosphate-buffered saline (PBS; Gibco, Life Technologies, Loughborough, UK) and cultivable bacterial species quantitated by plating serial dilutions on solid media and incubated under aerobic, microaerobic and anaerobic conditions (37 °C, 48 h) as stated elsewhere [30]. The numbers of respective bacteria were determined by enumeration of distinct colony morphotypes, followed by subcultivation, Gram-staining and biochemical analyses [30].

### 2.7. Culture-Independent Intestinal Microbiota Analysis

For quantitative assessment of fastidious and even uncultivable bacteria within bacterial suspensions and fecal samples, we applied culture-independent, 16S rRNA-based methods. The total genomic DNA was extracted from respective samples and adjusted to 1 ng per µL (Quant-iT PicoGreen reagent, Invitrogen, Carlsbad, CA, USA) as reported earlier [30]. The total eubacterial loads and the main bacterial groups abundant in the murine intestinal microbiota, including gamma-*Proteobacteria/*Enterobacteriaceae, *Enterococcus* genus, *Lactobacillus* group, *Bifidobacterium* genus, *Bacteroides* group, including *Prevotella* and *Porphyromonas*, *Clostridium coccoides* group, and *Clostridium leptum* group, were then assessed by quantitative RT–PCR (qRT–PCR) with species-, genera- or group-specific 16S rRNA gene primers (Tib MolBiol, Berlin, Germany), as shown in Table 1 and expressed as 16S rRNA gene copies per ng DNA [34].

### 2.8. Clinical Conditions

Before and once a day after the *C. coli* challenges, we quantitatively determined the clinical conditions of mice as stated previously [35]. In brief, the following parameters were assessed: the clinical aspect/wasting (0: normal; 1: ruffled fur; 2: less locomotion; 3: isolation; 4: severely compromised locomotion, pre-final aspect), the abundance of blood in feces (0: no blood; 2: microscopic detection of blood by the Guajac method using Hemoccult, Beckman Coulter/PCD, Krefeld, Germany; 4: macroscopic blood visible), and stool consistency (0: formed feces; 2: pasty feces; 4: liquid feces). The overall clinical score (maximum of 12) was calculated as the sum of the three scores assessing respective individual parameters [35].

### 2.9. Sampling Procedures

Mice were sacrificed by CO_2_ asphyxiation on day 6 p.i. Under sterile conditions, cardiac blood, large intestinal tissue samples (collected from each mouse in parallel for microbiological and immunohistopathological analyses) and luminal stomach, duodenum, ileum and colon samples were taken.

### 2.10. Histopathology

Histopathological changes were quantitated in 5 μm thin sections of colonic explants that had been immediately fixed in 5% formalin, embedded in paraffin and stained with hematoxylin and eosin using standardized histopathological (100× magnification, light microscopy, blinded investigator) as described earlier [36]. In brief, score 1: minimal inflammatory cell infiltrates in the mucosa with intact epithelium; score 2: mild inflammatory cell infiltrates in the mucosa and submucosa with mild hyperplasia and mild goblet cell loss; score 3: moderate inflammatory cell infiltrates in the mucosa with moderate goblet cell loss; score 4: marked inflammatory cell infiltration into in the mucosa and submucosa with marked goblet cell loss, multiple crypt abscesses and crypt loss.

### 2.11. In Situ Immunohistochemistry

In situ immunohistochemical analyses were performed in 5 μm thin large intestinal paraffin sections for quantification of apoptotic epithelial cells, macrophages and monocytes, T lymphocytes, regulatory T cells and B lymphocytes by using primary antibodies directed against cleaved caspase-3 (Asp175, Cell Signaling, Beverly, MA, USA, 1:200), F4/80 (no. 14-4801, clone BM8, eBioscience, San Diego, CA, USA, 1:50), CD3 (no. N1580, Dako, Glostrup, Denmark; 1:10), FOXP3 (clone FJK-165, no. 14-5773, eBioscience, 1:100) and B220 (no. 14-0452-81, eBioscience; 1:200), respectively [37]. The average number of positively stained cells in each section was determined within at least six high-power fields (HPF, 0.287 mm^2^, 400× magnification, blinded investigator).

### 2.12. Proinflammatory Cytokines

Ex vivo biopsies obtained from the colon and ileum (longitudinally cut strips of approximately 1 cm^2^) as well as from mesenteric lymph nodes (MLN; 3 lymph nodes) were washed in PBS (Gibco, Life Technologies, Loughborough, UK) and placed in 24-flat-bottom well culture plates (Nunc, Darmstadt, Germany) containing 500 μL serum-free RPMI 1640 medium (Gibco, Life Technologies, Loughborough, UK) supplemented with penicillin (100 µg/mL) and streptomycin (100 µg/mL; Biochrom, Berlin, Germany). Respective culture supernatants and serum samples were tested for interferon-γ (IFN-γ) and tumor necrosis factor-α (TNF-α) by the mouse inflammation cytometric bead assay (CBA; BD Biosciences, Heidelberg, Germany) in a BD FACSCanto II flow cytometer (BD Biosciences) after incubation at 37 °C for 18 h.

### 2.13. Statistical Analyses

Medians and significance levels were calculated by using GraphPad Prism (version 8, San Diego, CA, USA). The Mann–Whitney test was applied for pairwise comparisons of not normally distributed data. For multiple comparisons, the one-sided ANOVA with Tukey’s post-correction was used for normally distributed data and the Kruskal–Wallis test with Dunn’s post-correction for not normally distributed data. Two-sided probability (*p*) values ≤ 0.05 were considered significant. Definite outliers were removed after being identified by the Grubb’s test (α = 0.001). Data were pooled from three independent experiments.

## 3. Results

### 3.1. Gastrointestinal Pathogen Loads Following Oral Aviguard^®^ Treatment of C. coli Infected Secondary Abiotic IL-10^−/−^ Mice

Secondary abiotic IL-10^−/−^ mice were infected with 10^9^ viable *C. coli* cells on days 0 and 1 by gavage and perorally treated with the commercial competitive exclusion product Aviguard^®^ on days 2, 3 and 4 p.i. Our cultural analyses of the bacterial suspensions revealed total numbers of 10^9^ viable bacteria per mL, including 10^6^ CFU Enterobacteriaceae per mL and between 10^8^ and 5 ×10^8^ CFU *Lactobacillus*, *Enterococcus*, *Bacteroides/Prevotella* and *Clostridium/Eubacterium* species per mL (Figure S1A). We additionally performed culture-independent, 16S rRNA-based analyses for quantifying fastidious and non-cultivable bacteria. In support of the cultural data, our molecular analyses revealed consistently high gene numbers of Enterobacteriaceae, *Enterococcus* genus, *Lactobacillus* group, *Bifidobacterium* genus, *Clostridium coccoides* and *Clostridium leptum* groups among individual gavages (Figure S1B). Hence, the vast majority of cultivable, fastidious and uncultivable probiotic bacterial species abundant in the commercial competitive exclusion product Aviguard^®^ (except for *Fusobacterium* and *Propionibacterium* species) could be assessed by cultural and molecular approaches.

**Figure 1:**
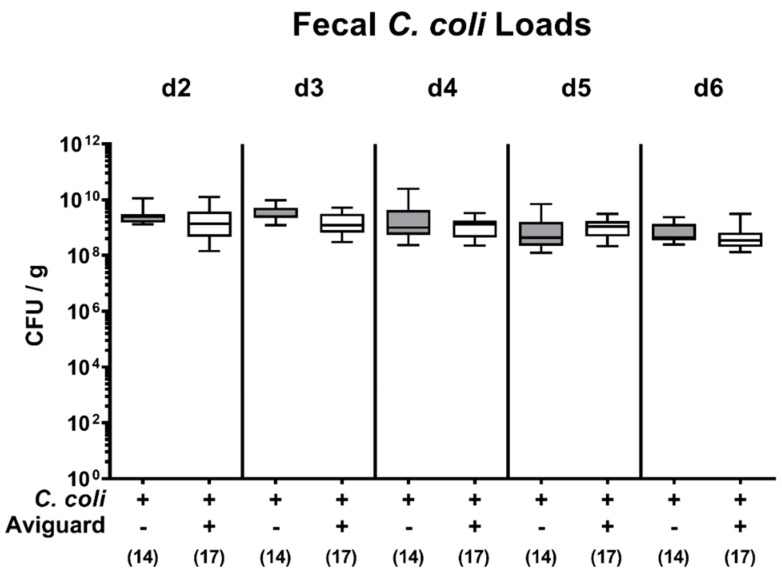
Comparative analyses of fecal pathogen loads following peroral application of placebo versus the probiotic formulation Aviguard^®^ to *C. coli*-infected secondary abiotic IL-10^−/−^ mice. Secondary abiotic IL-10^−/−^ mice were infected with a *C. coli* patient isolate on day (d) 0 and d1 by gavage. On d2, d3 and d4, post-infection mice were perorally challenged the commercial competitive exclusion product Aviguard^®^ (white boxes) or received placebo (gray boxes). The fecal *C. coli* loads were quantitatively assessed by culture (in colony-forming units per g, CFU/g). The box plots indicate the 25th and 75th percentiles of the medians (bar within boxes). The total range and the numbers of analyzed animals (in parentheses) are given. Pooled data were derived from three independent experiments. +, with; −, without.

Our cultural analyses revealed that *C. coli* could stably establish within the intestinal tract of both Aviguard^®^ and placebo-treated mice with comparably high median loads of 10^9^ CFU per g feces as early as day 2 p.i. (Figure 1, Figure S2). Upon necropsy on day 6 p.i., the pathogen numbers were slightly lower than day 2 p.i. in Aviguard^®^ treated mice (less than 0.5 logs; *p* < 0.05; Figure S2B), which also held for the placebo control mice, however (*p* < 0.01; Figure S2A). Hence, Aviguard^®^ could not sufficiently reduce fecal pathogen loads in *C. coli*-infected mice.

**Figure 2:**
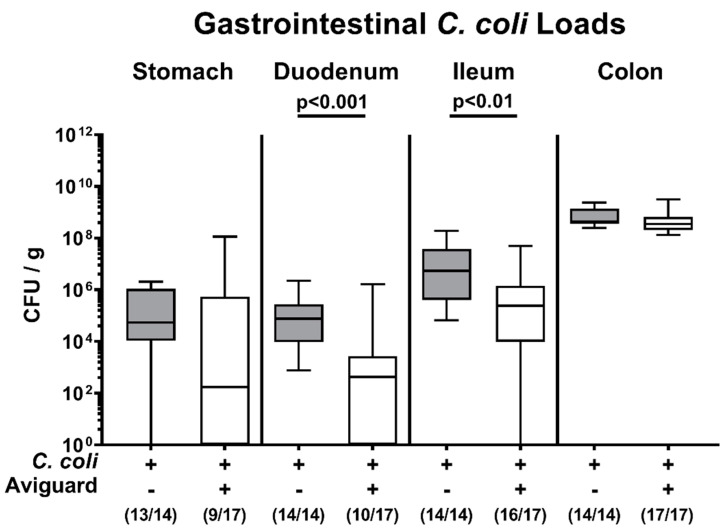
Gastrointestinal pathogen loads following peroral treatment of *C. coli*-infected secondary abiotic IL-10^−/−^ mice with the probiotic formulation Aviguard^®^. Mice were infected with a *C. coli* patient isolate on day (d) 0 and d1 by gavage. On d2, d3 and d4 post-infection (p.i.), mice were perorally challenged with the commercial competitive exclusion product Aviguard^®^ (white boxes) or received placebo (gray boxes). On d6 p.i., the pathogen loads were quantitatively assessed in distinct compartments of the gastrointestinal tract (indicated) by culture (in colony-forming units per g, CFU / g). The box plots indicate the 25th and 75h percentiles of the medians (bar within boxes). The total range, the significance levels (*p* values) calculated by the Mann–Whitney U test and the numbers of culture-positive mice out of the total number of analyzed animals (in parentheses) are given. Pooled data were derived from three independent experiments. +, with; −, without.

We further assessed the pathogen burdens in defined luminal parts of the gastrointestinal tract. On day 6 p.i., approximately two log orders of magnitude lower *C. coli* numbers could be determined in the duodenum and the ileum of Aviguard^®^ as compared to placebo-treated mice (*p* < 0.001 and *p* < 0.01, respectively; Figure 2), which was, however, not the case in the large intestines (Figure 2). Notably, 47.1% of mice from the Aviguard^®^ cohort, but only 7.1% of the placebo controls had expelled the pathogen from their stomach lumen. Furthermore, the former exhibited a trend towards approximately 2.5 log orders of magnitude lower median gastric *C. coli* numbers than the latter (not significant (n.s.) given high standard deviations; Figure 2). Hence, Aviguard^®^ treatment lowered the pathogen burdens within the proximal but not the distal intestinal tract of *C. coli*-infected IL-10^−/−^ mice.

### 3.2. Fecal Microbiota Composition Following Oral Aviguard^®^ Treatment of C. coli-Infected Secondary Abiotic IL-10^−/−^ Mice

**Figure 3:**
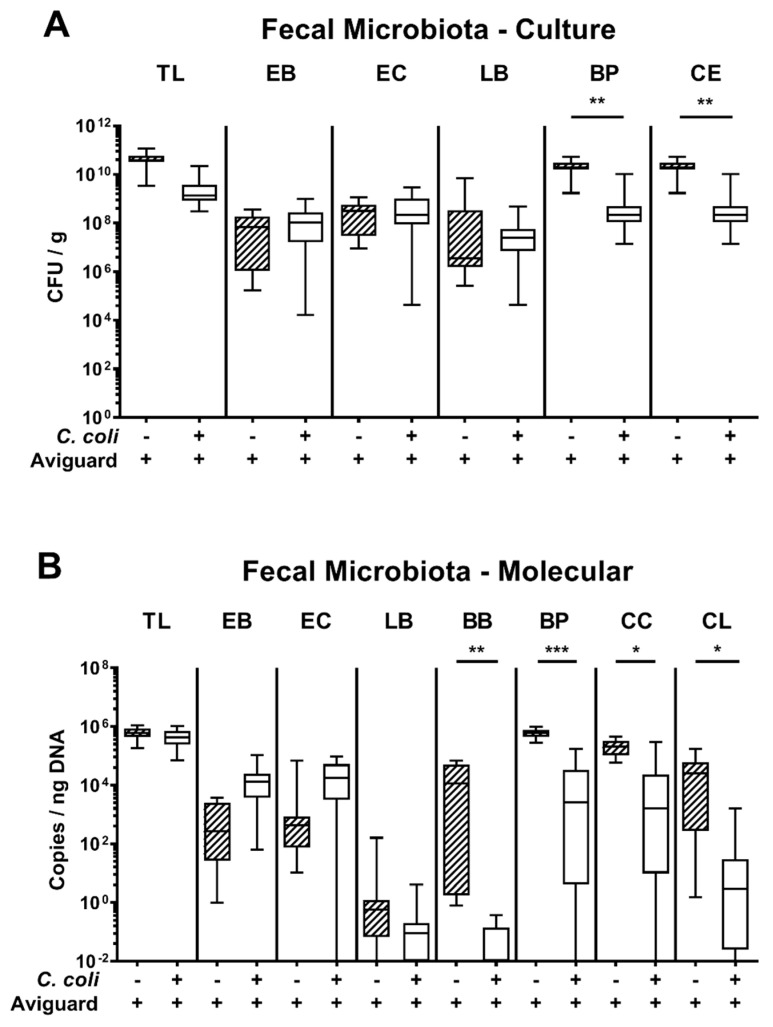
Comprehensive analysis of the gut microbiota composition following peroral Aviguard^®^ treatment of *C. coli*-infected secondary abiotic IL-10^−/−^ mice. Mice were infected with a *C. coli* patient isolate on the day (d) 0 and d1 by gavage and perorally challenged with the probiotic formulation Aviguard^®^ (*n* = 17) on d2, d3 and d4 post-infection (p.i.). On d6 p.i., the fecal microbiota composition was quantitatively surveyed by (**A**) culture (expressed as colony-forming units per g, CFU/g) and by (**B**) molecular (i.e., 16S rRNA based) methods (expressed as copies / ng DNA). Aviguard^®^ treated non-infected mice served as controls (*n* = 10). The box plots indicate the 25th and 75th percentiles of the medians (bar within boxes). Total range and significance levels (*p* values) calculated by the Mann–Whitney U test are given. Shown data were derived from three independent experiments. TL, total bacterial load; EB, Enterobacteriaceae; EC, *Enterococcus* genus; LB, *Lactobacillus* species; BP, *Bacteroides/Prevotella* genus; CE, *Clostridium/Eubacterium* species; BB, *Bifidobacterium* genus; CC, *Clostridium coccoides* group; CL, *Clostridium leptum* group.* *p* < 0.05; ** *p* < 0.01; *** *p* < 0.001. +, with; −, without.

We then addressed whether the applied Aviguard^®^ bacteria could stably establish within the intestinal tract of *C. coli*-infected and uninfected secondary abiotic IL-10^−/−^ mice and whether *C. coli* infection resulted in shifts within the gut microbiota composition. Our cultural analyses revealed that respective aerobic and anaerobic bacterial groups, genera and species could be detected in fecal samples obtained on day 6 p.i. with median loads ranging from approximately 10^6^ up to 10^11^ CFU per g. Interestingly, *C. coli*-infected mice harbored lower obligate anaerobic bacterial species, such as *Bacteroides/Prevotella* and *Clostridium/Eubacterium* species in their feces (*p* < 0.01, Figure 3A). In support, when applying culture-independent, 16S rRNA-based approaches, lower fecal gene numbers for *Bacteroides/Prevotella* group/genera (*p* < 0.001), for *Clostridium coccoides* (*p* < 0.05) and for *Clostridium leptum* groups (*p* < 0.05) as well as for *Bifidobacterium* genus (*p* < 0.01) were detected in *C. coli-*infected than uninfected mice following Aviguard^®^ challenge (Figure 3B). Hence, the probiotic bacteria could sufficiently establish in the intestinal tract of mice until day 6 p.i. Thus, 48 h after the latest of three consecutive peroral Aviguard^®^ applications with lower fecal loads of obligate anaerobic species in the *C. coli* cohort than the uninfected counterparts.

### 3.3. Clinical and Histopathological Sequelae Upon Oral Aviguard^®^ Treatment of C. coli-Infected Secondary Abiotic IL-10^−/−^ Mice

**Figure 4:**
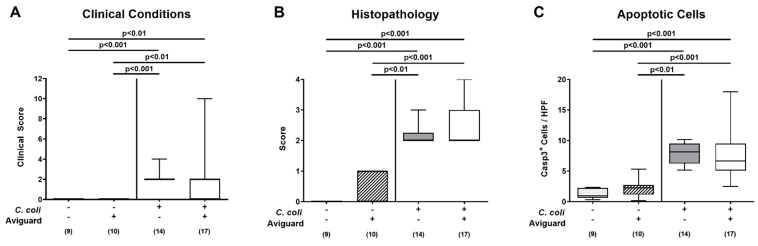
Clinical conditions, histopathological and apoptotic epithelial cell responses in the colon following peroral Aviguard^®^ treatment of *C. coli*-infected secondary abiotic IL-10^−/−^ mice. Mice were infected with a *C. coli* patient isolate on day (d) 0 and d1 by gavage. On d2, d3 and d4 post-infection (p.i.), mice were perorally challenged with the probiotic formulation Aviguard^®^ (white boxes) or received placebo (gray boxes). On d6 p.i., (**A**) the clinical condition of mice were quantified applying a clinical scoring system (see methods), and (**B**) histopathological changes in the colonic mucosa and lamina propria were assessed with standardized histopathological scores. Furthermore, the average numbers of colonic epithelial (**C**) apoptotic (Casp3 ^+^ ) cells were determined microscopically from six high-power fields (HPF, 400 × magnification) per mouse in immunohistochemically stained colonic paraffin sections. The box plots indicate the 25th and 75th percentiles of the medians (bar within boxes). Total range, significance levels (*p* values) calculated by the Kruskal–Wallis test and Dunn’s post-correction and numbers of analyzed mice (in parentheses) are given. Pooled data were derived from three independent experiments. +, with; −, without.

We further assessed the clinical outcomes of *C. coli*-infected IL-10^−/−^ mice following Aviguard^®^ treatment. Therefore, we quantified the key clinical signs of campylobacteriosis, such as wasting, the abundance of fecal blood and diarrhea in mice before and after *C. coli* infection by using standardized clinical scores [35]. Overall, mice from either cohort displayed rather mild clinical signs of *C. coli* infection as indicated by median scores not exceeding 2. Immediately before the first (i.e., day 2 p.i.) until the last (i.e., day 4 p.i.) of three consecutive Aviguard^®^ versus placebo applications, mice from the treatment cohort displayed slightly lower clinical scores than the placebo control group (*p* < 0.05–0.001; Figure S3), whereas at the end of the observation period (i.e., day 6 p.i.), the clinical conditions of *C. coli-*infected mice from either group were comparable (n.s.; Figure S3; Figure 4A).

We further quantitatively determined the large intestinal histopathological changes upon Aviguard^®^ treatment of *C. coli*-infected mice in colonic paraffin sections applying standardized histopathological scores [36]. On day 6 p.i., mild to moderate histopathological changes within the colonic mucosa could be observed (*p* < 0.01–0.001 versus uninfected controls), but with no differences between the Aviguard^®^ and the placebo cohorts (n.s.; Figure 4B).

Since apoptosis is well-known as a reliable parameter for the grading of intestinal inflammatory responses [31], we further enumerated colonic epithelial cells positive for cleaved caspase-3 following immunohistochemical staining of large intestinal paraffin ex vivo biopsies. In line with the obtained histopathological results, *C. coli* infection mounted in increased numbers of apoptotic colonic epithelial cells (*p* < 0.01–0.001), whereas comparable counts could be assessed in the Aviguard^®^ and placebo groups at day 6 p.i. (n.s.; Figure 4C). Hence, Aviguard^®^ did neither affect clinical signs of *C. coli* infection nor histopathological nor apoptotic changes in the large intestines of infected IL-10^−/−^ mice.

### 3.4. Intestinal and Systemic Proinflammatory Immune Responses Following Oral Aviguard^®^ Treatment of C. coli-Infected Secondary Abiotic IL-10^−/−^ Mice

**Figure 5:**
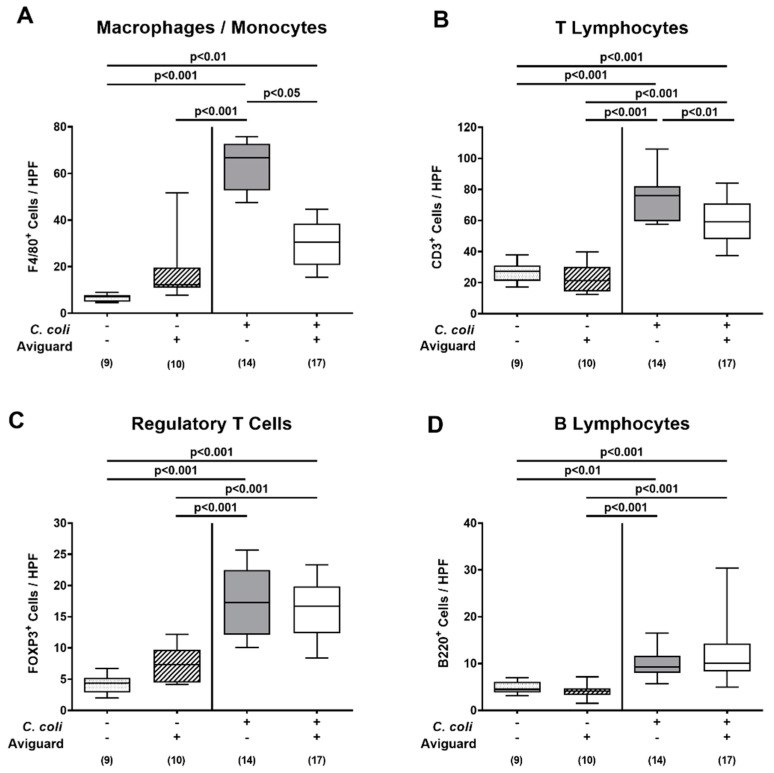
Colonic immune cell responses following peroral Aviguard^®^ treatment of *C. coli*-infected secondary abiotic IL-10^−/−^ mice. Mice were infected with a *C. coli* patient isolate on day (d) 0 and d1 by gavage. On d2, d3 and d4 post-infection (p.i.), mice were perorally challenged with the probiotic formulation Aviguard^®^ (white boxes) or received placebo (gray boxes). On d6 p.i., the average numbers of (**A**) macrophages and monocytes (F4/80 ^+^ ), (**B**) T lymphocytes (CD3 ^+^ ), (**C**) regulatory T cells (FOXP3 ^+^ ) and (**D**) B lymphocytes (B220 ^+^ ) were determined microscopically from six high-power fields (HPF, 400 × magnification) per animal in immunohistochemically stained colonic paraffin sections. The box plots indicate the 25th and 75th percentiles of the medians (bar within boxes). Total range, significance levels (*p* values) calculated by the ANOVA test with Tukey’s post-correction or the Kruskal–Wallis test and Dunn’s post-correction and numbers of analyzed mice (in parentheses) are given. Pooled data were derived from three independent experiments. +, with; −, without.

We further addressed whether peroral Aviguard^®^ application to *C. coli*-infected secondary abiotic IL-10^−/−^ mice modulated pathogen-induced immune responses in the intestinal tract. Therefore, we quantified distinct innate and adaptive immune cell populations in colonic paraffin sections that had been subjected to defined immunohistochemical stainings. On day 6 p.i., increased numbers of innate immune cells, such as macrophages and monocytes as well as of adaptive immune cell subsets, including T lymphocytes, regulatory T cells and B lymphocytes, could be determined in the colonic mucosa and lamina propria of mice from either cohort (*p* < 0.01–0.001; Figure 5). The *C. coli* induced increases in macrophages and monocytes as well as in T lymphocytes were, however, less pronounced following Aviguard^®^ than placebo treatment (*p* < 0.05 and *p* < 0.01 versus placebo, respectively; Figure 5A,B), whereas colonic numbers of regulatory T cells and B lymphocytes were comparable in both cohorts on day 6 p.i. (n.s.; Figure 5C,D).

**Figure 6:**
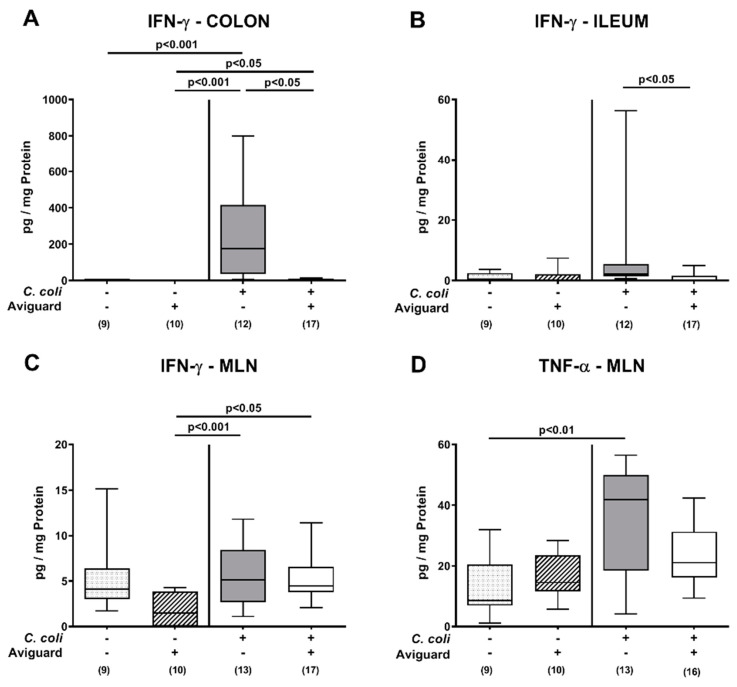
Intestinal secretion of proinflammatory cytokines following peroral Aviguard^®^ treatment of *C. coli-*infected secondary abiotic IL-10^−/−^ mice. Mice were infected with a *C. coli* patient isolate on day (d) 0 and d1 by gavage. On d2, d3 and d4 post-infection (p.i.), mice were perorally challenged with the probiotic formulation Aviguard^®^ (white boxes) or received placebo (gray boxes). On d6 p.i., intestinal IFN-γ concentrations were measured in ex vivo biopsies derived from the (**A**) colon, (**B**) ileum and (**C**) mesenteric lymph nodes (MLN). Furthermore, (**D**) TNF-α concentrations were determined in MLN. The box plots indicate the 25th and 75th percentiles of the medians (bar within boxes). Total range, significance levels (*p* values) calculated by the Kruskal–Wallis test and Dunn’s post-correction and numbers of analyzed mice (in parentheses) are indicated. Outliers were excluded after identification by the Grubb’s test (α = 0.001). Pooled data were derived from four independent experiments. +, with; −, without.

We further assessed proinflammatory cytokine secretion in distinct intestinal compartments. Elevated IFN-γ concentrations were measured in ex vivo biopsies taken from the colon of *C. coli*-infected mice (*p* < 0.05–0.001), but with lower levels in Aviguard^®^ than placebo-treated counterparts (*p* < 0.05; Figure 6A), whereas ileal IFN-γ secretion was less pronounced in the former versus the latter on day 6 p.i. (*p* < 0.05; Figure 6B). In MLN, increases in IFN-γ concentrations were comparable in both treatment groups on day 6 p.i. (*p* < 0.05–0.001; Figure 6C), while *C. coli*-induced TNF-α secretion was enhanced in placebo control mice only (*p* < 0.01; Figure 6D).

**Figure 7:**
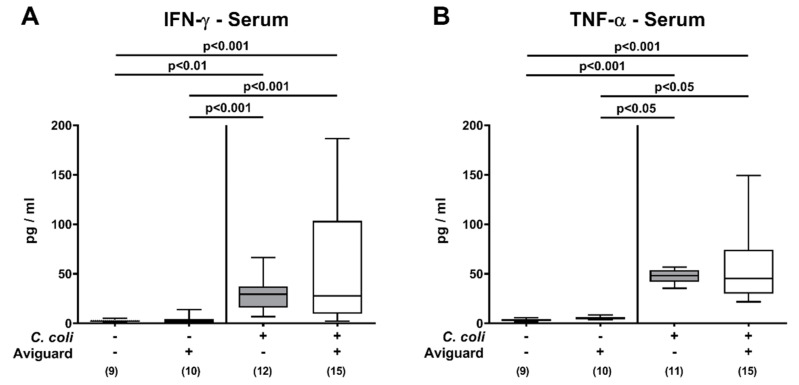
Systemic proinflammatory cytokine secretion following peroral Aviguard^®^ treatment of *C. coli*-infected secondary abiotic IL-10^−/−^ mice. Mice were infected with a *C. coli* patient isolate on day (d) 0 and d1 by gavage. On d2, d3 and d4 post-infection (p.i.), mice were perorally challenged with the probiotic formulation Aviguard^®^ (white boxes) or received placebo (gray boxes). On day 6 p.i., (**A**) IFN-γ and (**B**) TNF-α concentrations were measured in serum samples. The box plots indicate the 25th and 75th percentiles of the medians (bar within boxes). Total range, significance levels *(p* values) calculated by the Kruskal–Wallis test and Dunn’s post-correction and numbers of analyzed mice (in parentheses) are indicated. Outliers were excluded after identification by the Grubb’s test (α = 0.001). Pooled data were derived from four independent experiments. +, with; −, without.

We finally addressed whether the immuno-modulatory effects of Aviguard^®^ were restricted to the intestinal tract or could also be observed systemically. Therefore, we measured proinflammatory cytokines in serum samples taken in *C. coli*-infected and corresponding uninfected control groups. Upon *C. coli* infection, increased IFN-γ and TNF-α serum concentrations could be measured (*p* < 0.01–0.001) but did not differ between Aviguard^®^ or placebo-treated mice on day 6 p.i. (n.s.; Figure 7). Hence, Aviguard^®^ treatment of *C. coli*-infected IL-10^−/−^ mice dampened pathogen-induced proinflammatory immune responses in different compartments of the intestinal tract.

## 4. Discussion

In our actual study, we addressed to the best of our knowledge for the first time the impact of the oral treatment with the competitive exclusion product Aviguard^®^ in *C. coli*-induced murine campylobacteriosis. Following a 3-day oral treatment period starting two days after *C. coli* infection, Aviguard^®^ when compared to placebo challenged secondary abiotic IL-10^−/−^ mice i.) exhibited lower pathogen burdens within the proximal (i.e., duodenum, ileum), but not the distal intestinal tract (i.e., colon), ii.) presented with comparable pathogen-induced clinical signs, histopathological and apoptotic epithelial cell changes in the colon, whereas iii.) intestinal, but not systemic proinflammatory immune responses were dampened on day 6 p.i.

The decrease in pathogen loads by two log orders of magnitude within the small intestinal tract needs to be regarded as modest. One needs to take into consideration that the ecological niches taken by *Campylobacter* are predominantly the crypts within the large intestines [47,48]. In the colon, however, Aviguard^®^ could not exhibit pathogen-lowering effects until day 6 p.i. One may argue that (i) the oral infection dose of 10^8^ *C. coli* cells resulting in fecal and colonic pathogen loads of more than 10^8^ CFU per gram was too high (and beyond usual infection doses); and/or, (ii) the Aviguard^®^ treatment period was too short to achieve a biologically relevant pathogen-lowering effect and might hence have been more pronounced following longer application to mice or upon a prophylactic treatment starting prior *C. coli* infection, which we currently address in independent studies; (iii) furthermore, distinct bacterial components within the Aviguard^®^ formulation may either not have fully established within the gastrointestinal tract of the infected secondary abiotic murine host following peroral application. In earlier studies, applying a defined mucosal competitive exclusion flora before infection resulted in approximately two log orders of magnitude lower *C. jejuni* loads in the ceca of chickens following challenge with 10^5^ viable *C. jejuni* cells [49]. In line with a preceding trial by the same group applying standard competitive exclusion products [50], the authors questioned the relevant effectiveness of competitive exclusion products in reducing *C. jejuni* burdens in the avian host, which may result in the sustainable reduction of food safety risks as opposed to other enteropathogens, such as *Salmonella*, for instance [49,51].

Except for *Fusobacterium* and *Propionibacterium* genera that had not been included in our analytical panel, all other cultivable, fastidious and uncultivable probiotic bacterial species could be quantitatively assessed in the applied Aviguard^®^ suspension as well as in the intestinal luminal samples upon sacrifice by our comprehensive cultural and molecular analyses. Interestingly, colonic loads of obligate anaerobic bacterial taxa, including *Bacteroides/Prevotella*, *Clostridium* and *Bifidobacterium* species, were lower following Aviguard^®^ treatment of *C. coli*-infected than uninfected counterparts. In support, intestinal inflammatory conditions in mice and men are accompanied by pronounced shifts in the commensal gut microbiota composition as indicated by decreased diversity and particularly lower numbers of potentially probiotic species, such as *Bifidobacterium* species [52,53,54]. This further supports the pivotal triangle interactions between (entero)pathogens, commensal probiotic bacteria and host immunity during health and disease.

Apart from minor pathogen-lowering properties in the small intestines, oral Aviguard^®^ application exhibited potent immuno-modulatory, such as inflammation-dampening effects during acute murine campylobacteriosis. *C. coli-*infected mice that had been treated with the probiotic formulation displayed lower numbers of innate as well as adaptive immune cell subsets, such as macrophages, monocytes and T lymphocytes, respectively, infiltrating the infected large intestines which resulted in dampened secretion of proinflammatory cytokines in distinct parts of the intestinal tract, including the colon, ileum and MLN. However, the inflammation-alleviating effects were restricted to the intestines and could not be detected systemically given comparable proinflammatory cytokine concentrations in serum samples obtained from the Aviguard^®^ and placebo cohorts. Interestingly, lowered colonic T cell counts could be detected even in uninfected secondary abiotic wild-type mice until four weeks following three oral Aviguard^®^ applications [55].

In our previous *C. jejuni* infection studies, we showed that oral application of a single *Lactobacillus johnsonii* strain that had been isolated from the gut of a naive wild-type mouse [56] or the probiotic compound VSL#3 consisting of eight bacterial strains, including three *Bifidobacterium* species, four *Lactobacillus* species and *Streptococcus thermophilus* [57] could not effectively exclude *C. jejuni* from the already taken ecological niches within the large intestines of infected secondary abiotic wild-type mice. Instead, it effectively dampened the recruitment of macrophages, monocytes and T cells to the colonic mucosa and lamina propria [56,57], thus, further supporting the here obtained results following Aviguard^®^ treatment of *C. coli-*infected mice.

## 5. Conclusions

In summary, application of the probiotic formulation Aviguard^®^ to *C. coli*-infected mice does not result in a biologically relevant decrease in intestinal pathogen loads. However, it does modulate the host immune responses upon the infecting *C. coli* strain resulting in less distinct proinflammatory sequelae in the intestinal tract. Our study provides evidence that oral application of probiotic formulations, including Aviguard^®^, may be promising antibiotic-independent approaches to dampen *C. coli*-induced immune responses during human campylobacteriosis. Since the competitive exclusion products are usually applied in a prophylactic manner and not as treatment measures for already established enteropathogenic infection like in our survey, we currently evaluate the effects of Aviguard^®^ before *Campylobacter* infection.

## Figures and Tables

**Table 1:** 16S rRNA primer used for quantitative real-time-PCR ^a^.

Target	Reference Strain	Primer Sequence (5′ to 3′) and Orientation ^b^	Reference
Domain Bacteria (targets 16S V3 region)	*Escherichia coli* ATCC 25922	F:CGGYCCAGACTCCTACGGG, R:TTACCGCGGCTGCTGGCAC	[38]
γ-**Proteobacteria**/**Enterobacteriaceae**	*Escherichia coli* ATCC 25922	F: AAACTCAAATGAATTGACGG, R: CTTTTGCAACCCACTCC	[39]
*Enterococcus* genus	*Enterococcus faecalis* DSM 20478	F: CCTTATTGTTAGTTGCCATCATT, R: ACTCGTTGTACTTCCCATTGT	[40]
*Lactobacillus* group ^f^	*Lactobacillus acidophilus* DSM 20079	F: CACCGCTACACATGGAG, R: AGCAGTAGGGAATCTTCCA	[41,42]
*Bifidobacterium* genus	*Bifidobacterium* sp. (murine origin)	F: CTCCTGGAAACGGGTGG, R: GGTGTTCTTCCCGATATCTACA	[41,42,43]
Bacteroides group ^e^	*Bacteroides ovatus* DSMZ 1896	F: GAAGGTCCCCCACATTG, R: CAATCGGAGTTCTTCGTG	[44]
*Clostridium coccoides–Eubacterium rectale* subgroup ^d^	*Clostridium coccoides* DSMZ 935	F: AAATGACGGTACCTGACTAA, R: CTTTGAGTTTCATTCTTGCGAA	[43]
*Clostridium leptum* subgroup ^c^	*Clostridium leptum* DSMZ 753	F: TTACTGGGTGTAAAGGG, R: TAGAGTGCTCTTGCGTA	[45]

^a.^ according to reference [46] with minor modifications; ^b.^ F, forward; R, reverse; ^c.^, including *Faecalibacterium* (*Fusobacterium*) prausnitzii and *Clostridium* 16S rRNA cluster IV; ^d.^ *Clostridium* 16S rRNA cluster XIVa/b; ^e.^, including *Prevotella* and *Porphyromonas*; ^f.^, including *Leuconostoc*, *Pediococcus*, *Aerococcus* and *Weissella*, but not *Enterococcus* or *Streptococcus*; sp., species.

## Data Availability

The data presented in this study are available on request from the corresponding authors.

## Supplementary Materials

The following are available online at https://www.mdpi.com/article/10.3390/microorganisms9061127/s1, Figure S1: Microbiota composition of the perorally applied solutions containing the probiotic formulation Aviguard^®^, Figure S2: Fecal pathogen loads over time following peroral treatment of *C. coli*-infected secondary abiotic IL-10^−/−^ mice with the probiotic formulation Aviguard^®^, Figure S3: Clinical conditions over time following peroral treatment of *C. coli*-infected secondary abiotic IL-10^−/−^ mice with the probiotic formulation Aviguard^®^.
