# Supplementary material for: Survey of Pathogen-Lowering and Immuno-Modulatory Effects Upon Treatment of Campylobacter coli-Infected Secondary Abiotic IL-10−/− Mice with the Probiotic Formulation Aviguard®
Source: Microorganisms. 2021 May 23;9(6):1127. doi: 10.3390/microorganisms9061127 (PMC8224786; doi:10.3390/microorganisms9061127)
Supplement: Supplementary file 1 [file microorganisms-09-01127-s001.zip › Supplementary_FigureS1_06.04.21.pdf]

A Aviguard® Suspension - Culture

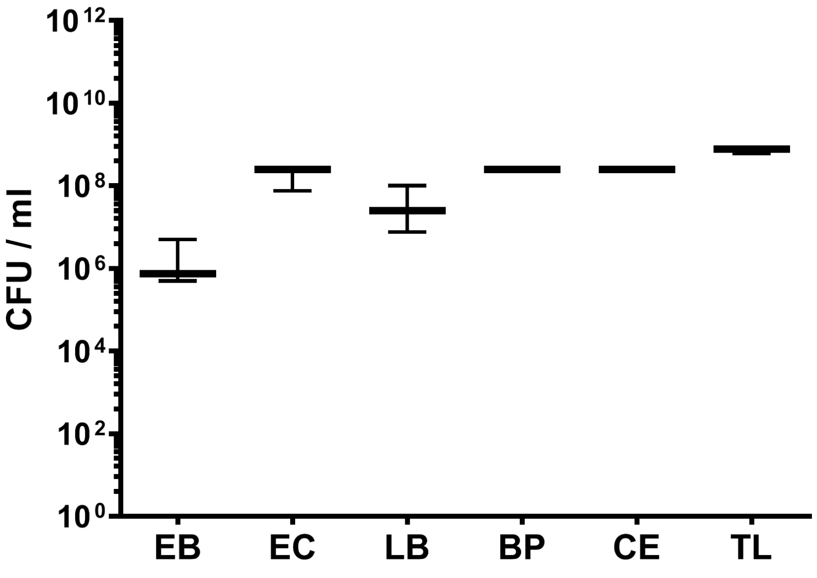

B Aviguard® Suspension - Molecular

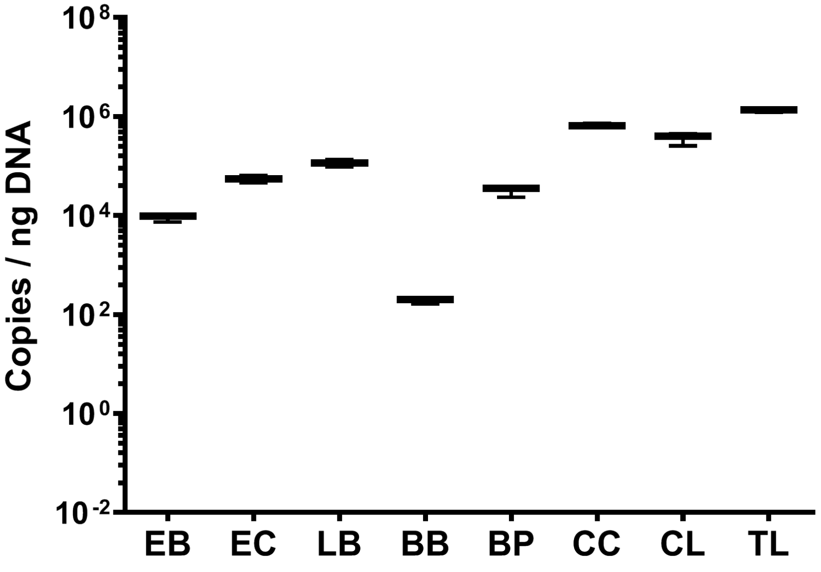

Supplementary Figure S1: Bacterial composition of Aviguard® suspensions used for the treatment of *C. coli* infected secondary abiotic IL-$10^{-/-}$ mice**.** Mice were infected with a *C. coli* patient isolate on day (d) 0 and d1 by gavage and perorally challenged with the commercial competitive exclusion product Aviguard® on d2, d3 and d4 post-infection (p.i.). The composition of the applied bacterial suspensions was quantitatively surveyed by **(A)** culture (expressed as colony forming units per g, CFU / g) and by **(B)** molecular (i.e., 16S rRNA based) methods (expressed as copies / ng DNA). The box plots indicating the 25th and 75th percentiles of the medians (bar within boxes) and the total ranges are given. Shown data were derived from three independent experiments. TL, total bacterial load; EB, *Enterobacteriacea*e; EC, *Enterococcus* genus; LB, *Lactobacillus* group; BP, *Bacteroides/Prevotella* group; CE, *Clostridium/Eubacterium* species; BB, *Bifidobacterium* genus; CC, *Clostridium coccoides* group; CL, *Clostridium leptum* group.
