# Supplementary material for: Survey of Pathogen-Lowering and Immuno-Modulatory Effects Upon Treatment of Campylobacter coli-Infected Secondary Abiotic IL-10−/− Mice with the Probiotic Formulation Aviguard®
Source: Microorganisms. 2021 May 23;9(6):1127. doi: 10.3390/microorganisms9061127 (PMC8224786; doi:10.3390/microorganisms9061127)
Supplement: Supplementary file 1 [file microorganisms-09-01127-s001.zip › Supplementary_FigureS2_06.04.21.pdf]

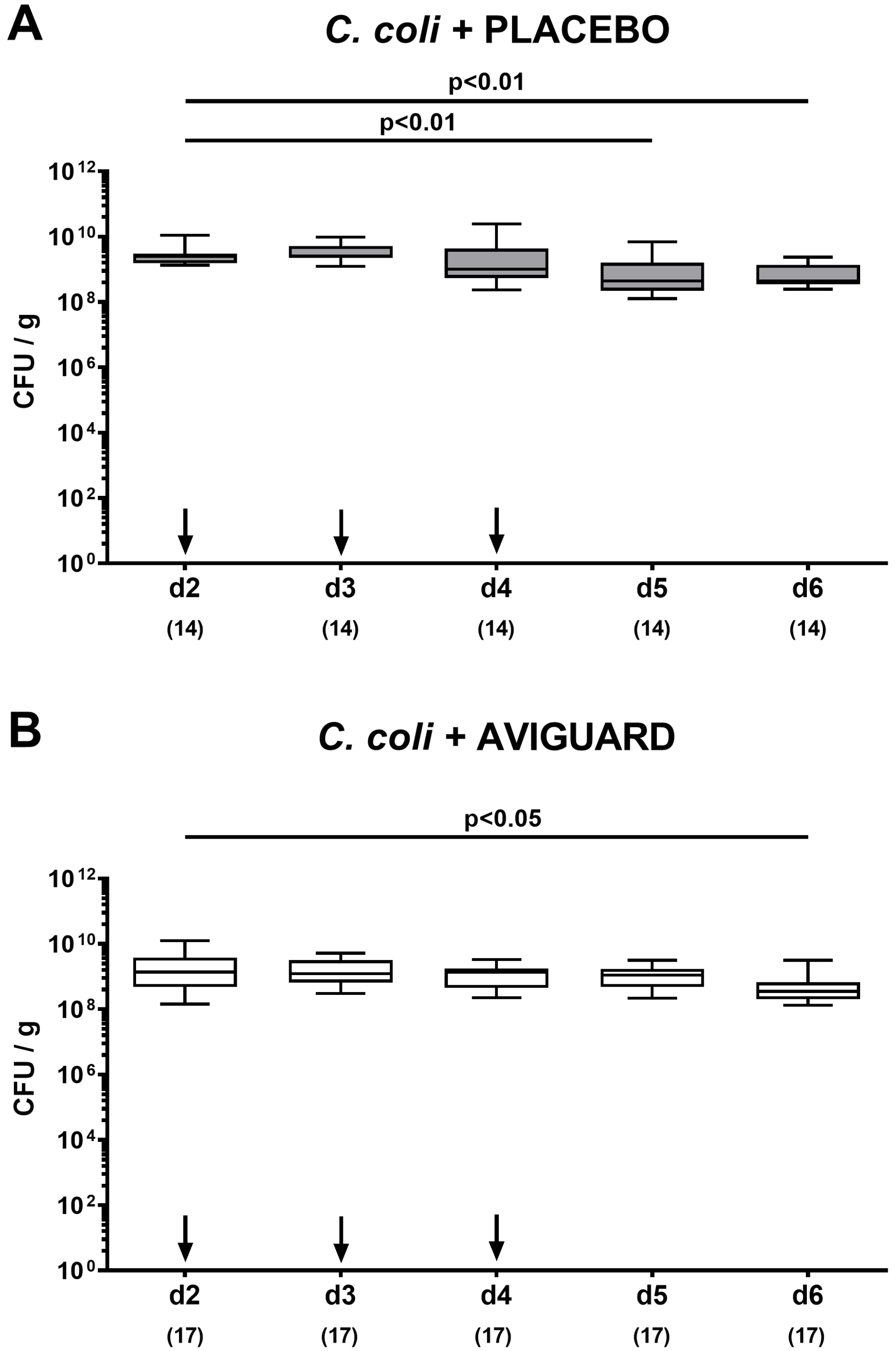

Supplementary Figure S2: Fecal pathogen loads over time following peroral application of placebo versus the commercial competitive exclusion product Aviguard® to *C. coli* infected secondary abiotic IL-$10^{-/-}$ mice. Secondary abiotic IL-$10^{-/-}$ mice were infected with a *C. coli* patient isolate on day (d) 0 and d1 by gavage. On d2, d3 and d4 post-infection (arrows) mice were perorally challenged with either **(A)** placebo (grey boxes) or **(B)** with the commercial competitive exclusion product Aviguard® (white boxes). The fecal *C. coli* loads were quantitatively assessed by culture (in colony forming units per g, CFU / g). The box plots indicate the 25th and 75th percentiles of the medians (bar within boxes). The total ranges, the significance levels (p values, versus d2) calculated by the Mann Whitney U test and the numbers of analyzed mice (in parentheses) are given. Shown data were derived from three independent experiments.
