# Supplementary material for: Survey of Pathogen-Lowering and Immuno-Modulatory Effects Upon Treatment of Campylobacter coli-Infected Secondary Abiotic IL-10−/− Mice with the Probiotic Formulation Aviguard®
Source: Microorganisms. 2021 May 23;9(6):1127. doi: 10.3390/microorganisms9061127 (PMC8224786; doi:10.3390/microorganisms9061127)
Supplement: Supplementary file 1 [file microorganisms-09-01127-s001.zip › Supplementary_FigureS3_06.04.2021.pdf]

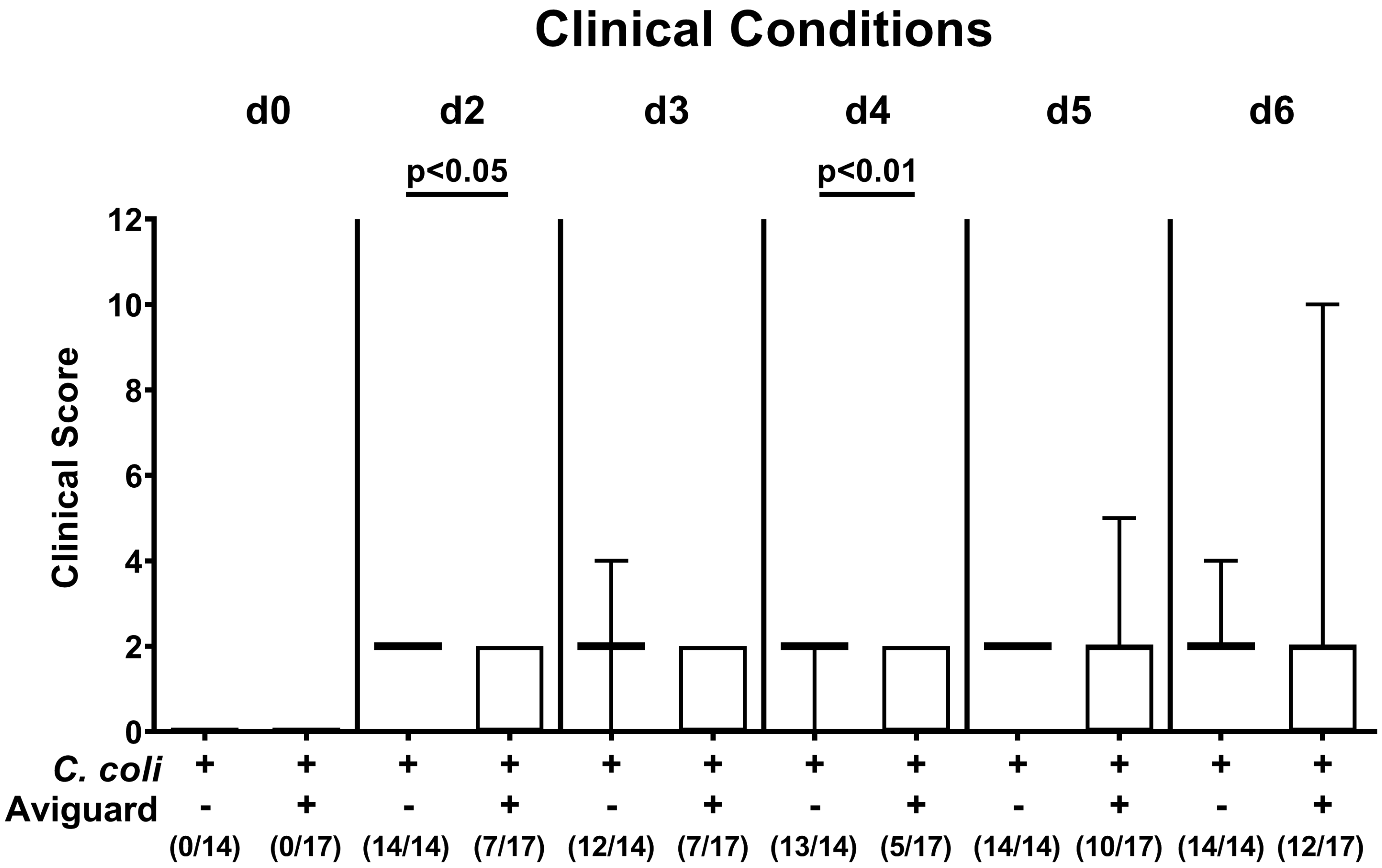

Supplemental Figure S3: Clinical conditions following peroral Aviguard® treatment of *C. coli* infected secondary abiotic IL-10$^{-/-}$ mice. Mice were infected with a *C. coli* patient isolate on day (d) 0 and d1 by gavage. On d2, d3 and d4 post-infection mice were perorally challenged with the commercial competitive exclusion product Aviguard® (white boxes) or received placebo (grey boxes). Immediately before and at defined time points after either peroral challenge, the clinical conditions of mice were quantitatively assessed applying standardized clinical scores (see methods). The box plots indicate the 25th and 75th percentiles of the medians (bar within boxes). The total ranges, the significance levels (p values) calculated by the Mann Whitney U test and the numbers of clinical score-positive mice out of the total number of analyzed animals (in parentheses) are indicated. Pooled data were derived from three independent experiments.
